# *DMRT3* Allele Frequencies in Batida- and Picada-Gaited Donkeys and Mules in Brazil

**DOI:** 10.3390/ani13243829

**Published:** 2023-12-12

**Authors:** Mariana Herman, Amanda Manara Caceres, Ana Luísa H. Albuquerque, Raíssa O. Leite, César Erineudo T. Araújo, Diego José Z. Delfiol, Rogério A. Curi, Alexandre S. Borges, José P. Oliveira-Filho

**Affiliations:** 1 Department of Veterinary Clinical Science, School of Veterinary Medicine and Animal Science, São Paulo State University (Unesp), Botucatu 18618-681, Brazil; mariana.herman@unesp.br (M.H.); amandamanarac@gmail.com (A.M.C.); luisahdealbuquerque@gmail.com (A.L.H.A.); raissa.leite@unesp.br (R.O.L.); alexandre.s.borges@unesp.br (A.S.B.); 2 School of Veterinary Medicine, Centro Universitário Doutor Leão Sampaio (Unileão), Juazeiro do Norte 63041-140, Brazil; cesararaujovet@hotmail.com; 3 School of Veterinary Medicine, Federal University of Uberlandia, Uberlandia 38405-314, Brazil; djzdelfiol@ufu.br; 4 Department of Breeding and Animal Nutrition, School of Veterinary Medicine and Animal Science, São Paulo State University (Unesp), Botucatu 18618-681, Brazil; rogerio.curi@unesp.br

**Keywords:** equids, gait keeper, locomotion pattern, genotype effect, Brazil

## Abstract

**Simple Summary:**

In Brazil, the production of gaited mules has been a prominent activity in agribusiness. The selection of gaited saddle mules with a comfortable gait for covering long distances at low speeds involves crossing marching donkeys of the Pêga breeds with horses, preferably those belonging to the Mangalarga Marchador and Campolina breeds. The reference-C and non-reference-A alleles of the *DMRT3*:g.22999655C>A SNP are linked with different horse gaits, including the batida gait (diagonalized) and the picada gait (lateralized) in Mangalarga Marchador and Campolina horses, respectively. Since donkeys (*Equus asinus*) and mules *(E. asinus* ♂ x *E. caballus* ♀) also exhibit these gaits, this study aimed to determine whether the genotype affects the gait type in these animals. The higher frequency of CA mules and the rare presence of the A allele of *DMRT3* in donkeys match previous findings in Mangalarga Marchador and Campolina horses, which are crucial in creating marching mules in Brazil. This suggests that the A allele likely came from the mares used in mating with donkeys. Furthermore, our findings suggest that factors beyond this gene variant, such as other genes and genetic variations, play a role in gait characteristics in equids.

**Abstract:**

In Brazil, the production of mules with a comfortable gait primarily involves the breeding of marching saddle mules. This is achieved by crossing gaited Pêga donkeys with horses from the Mangalarga Marchador and Campolina breeds. The *DMRT3*:g.22999655C>A SNP is implicated in regulating gait phenotypes observed in various horse breeds, including the batida (CC) and picada (CA) gaits found in these horse breeds. We aimed to determine if genotypes influenced gait type in 159 mules and 203 donkeys genotyped for the *DMRT3* SNP by PCR-RFLP analysis. About 47% of mules had the CC-genotype, while 53% had the CA-genotype. Donkeys predominantly had the CC-genotype (97%), and none had AA. Both CC- and CA-genotypes were evenly distributed among mules with the batida or picada gaits. In donkeys, the CC-genotype frequencies were consistent regardless of gait type. However, the CA-genotype was more common in picada-gaited donkeys than in batida-gaited donkeys. The prevalence of CA mules and the rare presence of the non-reference allele in donkeys align with previous findings in Mangalarga Marchador and Campolina horses. This suggests that the non-reference allele likely originated from the mares involved in donkey crosses. Our results also imply that factors beyond this variant, such as other genes and polymorphisms, influence gait traits in equids.

## 1. Introduction

*DMRT3 (doublesex and mab-3-related transcription factor 3*) is one of the main genes involved in vertebrate coordination of the front and hind limbs and control of stride length during locomotion [1,2,3,4,5]. A nonsense variant (g.22999655C>A, EquCab3.0) in the *DMRT3* gene, which is responsible for the stop codon (*DMRT3*_Ser301STOP) and results in the production of a truncated protein that is 174 amino acids shorter than the wild-type protein, has an important effect on domestic horse diversification by determining the gait phenotypes in different horse breeds [1].

Regarding worldwide frequency distribution, the genotyping of 4396 horses for the *DMRT3* variant confirmed a global distribution of this variant, as it was identified in 68 out of the 141 genotyped horse breeds to date [2]. This variant’s distribution worldwide has also been substantiated in other studies [6,7,8,9,10], with the highest frequency of polymorphism (AA) observed in breeds of horses either classified as gaited or as bred for harness racing [2]. A previous study indicated that Finnhorses with the AA genotype exhibited significantly better performance in harness racing than horses with the CA or CC genotypes. However, in the context of riding, it was evident that AA horses faced more challenges with the trot and canter gaits than CA and CC horses [11]. Conversely, the AA genotype did not demonstrate an association with superior performance, either early or late, in the Swedish-Norwegian Coldblooded Trotter breed used for harness racing [12]. Compared with the CA genotype, the AA genotype reinforces the coordination of ipsilateral legs, with a subsequent negative effect on the synchronized movement of contralaterally diagonal legs [3]. Several studies have associated the presence of two mutated alleles (AA) with gaited horses [2,3,4,5,7]. An evaluation of free-ranging Icelandic gaited horses concluded that while horses with the CC genotype could exhibit the gait known as tölt, horses with mutated alleles displayed a greater ease in lateralized movement to perform tölt. Furthermore, these horses demonstrated this movement at a higher frequency than unaffected animals [4]. The AA genotype also has a negative effect on characteristics associated with running [13,14].

Two gait types, batida and picada, have been established by the Mangalarga Marchador horses and the Pêga Donkey Breeders’ associations [15]. In the batida gait, the horse’s contralaterally diagonal hooves touch the ground in coupled steps more frequently than occurs with the lateral pairs of hooves, although moments of triple-limb support exist. In the picada gait, the steps are more often laterally coupled rather than being coupled in a contralaterally diagonal manner, all without loss of triple support moments [16]. Studies carried out on the Brazilian Mangalarga Marchador revealed a predominance of C and A alleles in horses that presented batida and picada gaits, respectively [16,17,18].

In Brazil, the production of gaited mules, which are obtained mainly from the crossing of gaited donkeys of the Pêga and Brasileira breeds with horses of the Mangalarga Marchador and Campolina breeds [19], has been a prominent activity in agribusiness, primarily for the commercial value of these animals that increasingly gain esteem in gait and morphological-functional sports contests. Given the insights from previous studies [17,18] that demonstrated the prevalence of the C and A alleles in Mangalarga Marchador horses associated with batida and picada gaits, respectively, it is noteworthy to consider that the gaits of donkeys (*E. asinus*) and mules (*E. asinus* ♂ x *E. caballus* ♀) are also classified as batida or picada and consequently could also be influenced by the *DMRT3* nonsense variant. This study aims to determine the allele frequencies of the *DMRT3* SNPs in gaited donkeys and mules and to verify whether the genotype influences the type of gait—batida or picada—in these animals, as has been proven in Mangalarga Marchador and Campolina horses [17,18].

## 2. Materials and Methods

### 2.1. Sample Collection

The sample size was calculated using OpenEpi software (version 3.0.1). Based on the number of donkeys (30,020) and mules (10,010) registered in the Associação Brasileira dos Criadores de Jumento Pêga (ABCJPÊGA), an estimated prevalence of *DMRT3* SNP of 5%, and a 5% margin of error. The results were a minimal sample size of 126 donkeys and 113 mules with a 99% confidence interval. A total of 203 Pêga male donkeys (*Equus asinus*) and 159 female mules (*E. asinus* ♂ x *E. caballus* ♀) were assessed in this study. Samples were obtained from 38 farms in the Southeast and Midwest regions of Brazil. The sampling was performed under a strict confidentiality agreement to ensure the anonymity of establishments, owners, and animals. The researchers tried to collect as many samples as possible according to the availability of the owners during their visits to the farms. All animals were classified by gait type, picada or batida, by an experienced technician according to ABCJPÊGA’s rules. Genomic DNA was purified from hair bulbs (from the mane or tail) or blood samples and stored frozen at −80 °C until molecular processing was conducted.

### 2.2. Sequencing of the Donkey DMRT3 Gene

We aimed to sequence the genomic DNA region corresponding to the coding sequence of the donkey *DMRT3* gene. To sequence the two exons from the donkey *DMRT3* gene, DNA samples from 20 Pêga male donkeys (10 with batida and 10 with picada gaits) were used. To include greater genome diversity in the analysis, the selected animals were unrelated to each other. Polymerase chain reaction (PCR) was performed using specific primers designed with the PrimerQuest™ Tool (Integrated DNA Technologies, Inc., Coralville, IA, USA) to amplify the complete coding *DMRT3* sequence (two exons) (XM_044756984.1) and the 5′ and 3′ intron–exon junctions (Table 1). The PCR mix (25 μL) contained 2.5 μL (200 ng) of template DNA, 0.4 μM of each primer, 12.5 μL of PCR Master Mix (Promega, San Luis Obispo, CA, USA), and 8.5 μL of nuclease-free water. The amplification conditions were as follows: initial denaturation at 95 °C for 5 min, followed by 40 cycles of denaturation at 95 °C for 45 s, annealing at 59–64 °C for 60 s, and a final extension at 72 °C for 60 s, with a final extension at 72 °C for 5 min.

The PCR products were analyzed by 1.5% agarose gel electrophoresis. The products with the correct size were purified using the GenElute^TM^ PCR Clean-Up Kit (Sigma-Aldrich^®^, St. Louis, MA, USA), according to the manufacturer’s instructions. To sequence the DNA, we used 10 μL of each purified PCR product, 5 μL of the forward primers (Table 1), and the BigDye^®^ Terminator Cycle Sequencing Kit (Life Technologies^TM^, Carlsbad, CA, USA). The obtained sequences and electropherograms were examined using Geneious^®^ 10.0 software (Biomatters^©^, Auckland, New Zealand) and were compared with the *Equus caballus* (horse) *DMRT3* coding sequence (GenBank^TM^ NC_009166.3:22378399-22378896) and the *Equus asinus DMRT3* coding predicted sequence (XM_044756984.1).

### 2.3. DMRT3 Genotyping Using PCR-RFLP Analysis

The *DMRT3*:g.22999655C>A SNP was genotyped in donkey and mule DNA samples using the polymerase chain reaction-restriction fragment length polymorphism (PCR-RFLP) method. Initially, DNA samples from Mangalarga Marchador horses were genotyped for the nonsense variant *DMRT3* by Sanger sequencing to identify the animals of the different genotypes (CC, CA, and AA). The control samples for each genotype were used for the standardized PCR-RFLP method.

Genomic DNA was purified from hair bulb samples using an in-house method based on Zabek et al. [20]. Specifically, DNA was obtained from 30 hair bulbs through overnight proteinase K digestion at 56 °C in lysis buffer K (composed of 10 mM Tris-HCl pH 8.3, 50 mM KCl, and 0.5% Tween 20). Proteinase K was inactivated by incubating the DNA samples at 95 °C for 10 min. For the DNA extracted from blood samples, the GenElute™ Genomic Blood DNA Kit (Sigma-Aldrich^®^) was utilized following the manufacturer’s instructions. Finally, all DNA samples were stored frozen at −20 °C.

PCR (25 μL) was performed with 2.5 μL (200 ng) of template DNA, 0.4 μM of each *DMRT3*_LBMCV_DdeI set primer, 12.5 μL of PCR Master Mix (Promega, CA, USA), and 8.5 μL of nuclease-free water. The amplification conditions were as follows: initial denaturation at 95 °C for 5 min, followed by 40 cycles of denaturation at 95 °C for 45 s, then annealing at 60 °C for 60 s, and extension at 72 °C for 60 s, and a final extension at 72 °C for 5 min. Immediately afterwards, for rapid detection of the *DMRT3*_g.22999655C>A SNP, the PCR products were cleaved using DdeI restriction enzymes (Promega, CA, USA). The RFLP reaction mixtures contained 7.3 μL of nuclease-free water, 10 μL of PCR product, 0.2 μL of bovine serum albumin, 2 μL of 10X reaction buffer, and 5 u of DdeI enzyme. Reactions were carried out at 37 °C for 30 min. The PCR amplicons and the products of restriction enzyme cleavage were analyzed by 2% agarose gel electrophoresis stained with GelRed^®^ Nucleic Acid Stain (Millipore^®^, Darmstadt, Germany). Sanger sequencing was performed for the *DMRT3* SNP to validate the PCR-RFLP results using PCR products before they were cleaved.

### 2.4. Data Analysis

The allele frequency and standard error for each group were estimated using the following equations: $\mathrm{allele}\;\mathrm{frequency}=\frac{\mathrm{total}\;\mathrm{number}\;\mathrm{of}\;\mathrm{mutant}\;\mathrm{alleles}\;/\mathrm{total}\;\mathrm{number}\;\mathrm{of}\;\mathrm{animals}}{2}$ and $\mathrm{standard}\;\mathrm{error}\;=\sqrt[{2}]{\frac{\mathrm{allele}\;\mathrm{frequency}\;*\;\left(1-\;\mathrm{allele}\;\mathrm{frequency}\right)}{2\;*\;\mathrm{sample}\;\mathrm{size}}}$. 

All calculations were performed using a spreadsheet program, as previously described [21]. The relationship between genotypes and gait types was analyzed using the chi-square test. Statistical significance was determined by a *p* ≤ 0.05, and the data analysis was performed using GraphPad Prism 7 software. The chi-square test was also used to test whether alleles were in Hardy-Weinberg equilibrium (HWE) within the donkey group, and the alleles were considered to exhibit disequilibrium if *p* < 0.05.

### 2.5. Limitations of the Study

The main limitations of this study were the impossibility of genotyping the mares used in the production of the mules and thus the impossibility of obtaining a reliable genealogy of these mules. It was not possible to access the genealogical data of the donkeys, so unfortunately, we were not able to calculate the degree of inbreeding of the studied animals. The lack of analysis of the *DMRT3* gene coding sequence in the mules may be seen as a limitation of this study; however, this did not fall under the objective of this study since these infertile hybrid animals cannot be used in inbreeding programs, and genotyping these animals would therefore not be a useful procedure in the design of mating schemes between donkeys and mares.

## 3. Results

The *DMRT3* mRNA sequence obtained from the Pêga donkey DNA samples was deposited in GenBank^TM^ (OP068195.1). Of the 1718 bases sequenced, 38 were from the 5′ UTR, 1425 bases were from the open reading frame (ORF; 460 bases in exon 1 and 965 bases in exon 2), and 255 bases were from the 3′ UTR. The BLAST algorithm revealed that this sequence was 99.94% and 99.59% similar to *Equus caballus DMRT3* mRNA (NM_001317265.1) and to that of the predicted *Equus asinus DMRT3* mRNA (XM_044756984.1), respectively. Six synonymous SNPs were observed in the donkey *DMRT3* coding sequence compared to that of the horse sequence, i.e., c.396 (C or T), p.132ALA; c.627 (C or G), p.209Val; c.966 (A or C), p.322Ser; c.1131 (T or C), p.377Tre; c.1137 (A or G), p.379Ala; and c.1251 (G or A), p.417Ser. One SNP (A/G) was also observed in exon 1, five bases before the start codon in the 5′ UTR. Furthermore, the predicted donkey *DMRT3* sequence has two synonymous SNPs (c.315T>C, p.105Ala; c.336C>G, p.112Pro) and three deleted bases (c.359_361delCGC) relative to the horse and Pêga donkey *DMRT3* coding sequences. The three protein sequences were 100% identical except for the deleted codon, p.Pro120del, in the predicted donkey *DMRT3* sequence. No changes were observed in the intronic region after exon 1 (146 bases) or before exon 2 (150 bases). To ensure consistency and prevent misinterpretations, we opted to use the same *DMRT3*_SNP nomenclature employed in prior equine studies (EquCab3.0 DMRT3_chr23:g.22999655C>A) rather than relying on the *E. asinus* sequence previously deposited in GenBank^TM^ (XM_044756984.1 c.902 C>A).

**Figure 1:**
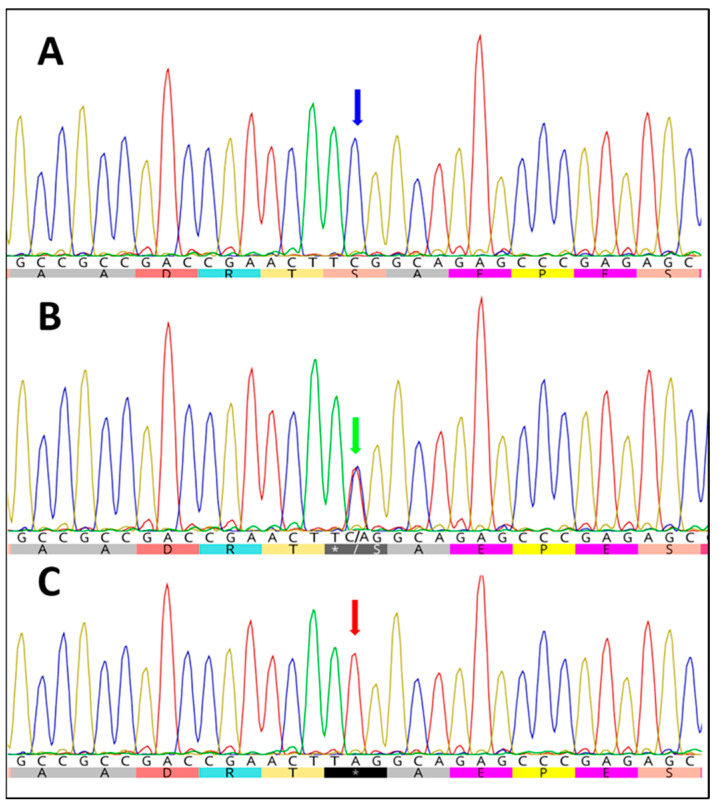
Partial chromatogram showing capillary sequencing results for wild-type homozygous (**A**), heterozygous (**B**), and mutated homozygous (**C**) EquCab3.0 *DMRT3*_chr23:g.22999655C>A variant (OP068195.1 c.902 C>A). Wild-type allele (cytosine) and the corresponding amino acid serine (blue arrow); double peaks (cytosine/guanine) and the corresponding amino acid stop codon or serine (green arrow) Mutant allele (adenine); and TAG stop codon (red arrow). Images were obtained using Geneious^®^ 10.0 software (Biomartters Ltd., Auckland, New Zealand).

Since the three genotypes had already been described in Mangalarga Marchador horses, we decided to use DNA samples from horses of this breed to standardize the PCR-RFLP method. Using the restriction enzyme DdeI, it was possible to use electrophoresis to identify the three genotypes, i.e., wild-type homozygous (CC, 560-bp band), mutant homozygous (AA, 395- and 165-bp bands), and heterozygous animals (CA, 560-, 395-, and 165-bp bands). These genotypes were confirmed by Sanger sequencing (Figure 1).

Of the 159 mules assessed, 47% (75/159) were homozygous wild-type (CC), and 53% (84/159) were heterozygous (CA) animals. The CC genotype was predominant in the Pêga donkeys (97%, 196/203), while seven (3%, 7/203) donkeys were identified as heterozygous (CA) for the *DMRT3* SNP. The AA genotype was not found in the mules or donkeys in this study (Table 2). Therefore, the C allele was the most frequent allele in the mules and donkeys evaluated (0.736 ± 0.025, 0.983 ± 0.006), while the frequencies of the A allele in the group of mules and donkeys evaluated, regardless of gait, were 0.264 (±0.025) and 0.017 (±0.006), respectively. The allele distribution of the *DMRT3* SNP in the donkey group was in HWE equilibrium (*p* = 0.879).

The mules and donkeys were grouped according to their gait type, with the result that 99 mules were classified as having the batida gait and 60 as having the picada gait, while 101 donkeys were classified as having the batida gait and 102 as having the picada gait (Table 3). Assessing the mules according to gait, the CC and CA genotypes were similarly distributed in the mule group with the batida gait (*p* = 0.484), as well as in the mule group with the picada gait (*p* = 0.749). When verifying the distribution of the CC and CA genotypes in the donkeys according to their gait phenotype the CC genotype was more predominant than the CA genotype in both the batida gait group (*p* < 0.001) and the picada gait group (*p* < 0.001). In addition, the prevalence of the CC genotype was statistically similar (*p* = 0.905) between the two gaits: batida (100/196, 51%) or picada (96/196, 49%). However, the CA genotype was statistically more associated (*p* < 0.001) with donkeys that had the picada gait (6/7, 86%) than with those that had the batida gait (1/7, 14%).

## 4. Discussion

The donkey (GenBank^TM^ OP068195.1) and horse *DMRT3* gene sequences are orthologues, given that in the donkey sequence, five synonymous SNPs in the coding region (c.627C>G, c.966A>C, c.1131T>C, c.1137A>G, and c.1251G>A) were observed; that is, these SNPs reflect no changes from the amino acid sequence previously described in horses (p.209Val, p.322Ser, p.377Tre, p.379Ala, p.417Ser). The *Equus caballus* sequence (GenBank^TM^ NC_009166.3:22378399-22378896) that we compared with the *Equus asinus* sequence was obtained through automated prediction, so it, cannot be said that the SNPs observed in the present study do not also occur in horses. The C allele of the synonymous SNP (c.966A>C, p.322Ser) was also observed in French (Poitu) donkeys and in two breeds of Indian donkeys (Spiti and Leh breeds). On the other hand, the Halari donkeys had only the A allele [22]. The A allele was observed in heterozygosis in a single donkey (1/30) of the group of Pega donkeys evaluated in the present study.

As the *DMRT3*_chr23:g.22999655C>A SNP is involved in the coordination of forelimbs and pelvic limbs during locomotion [1,4], some authors have standardized the genotyping of horses for that aforementioned polymorphism using different techniques, e.g., the TaqMan SNP probe [1,2,4], PCR-sequencing [17], mutagenically separated PCR (MS-PCR) [23], and PCR-RFLP [15,16,24]. As with previous studies [15,16,24] that employed the restriction enzyme DdeI, the methods of the present study entailed standardizing the PCR-RFLP in mules using specific sets of primers and the DdeI enzyme. This method enabled differentiation of the evaluated genotypes and was validated by Sanger sequencing, demonstrating its utility as an inexpensive and fast test for mutated alleles in mules and donkeys, thereby providing guidance for breeding strategies that favor the choice of the desired gait. 

Members of the Equidae family have different gaits and variations of these gaits (e.g., picada, batida, and the gaits of different gaited horse breeds) [17]. Using the ABCJPêga rules, the mules and donkeys of the present study were grouped according to their natural batida or picada gait pattern, which is to say that the interference of the human rider or trainer with the gait was not taken into account since mules may adapt to the type of gait needed by the trained rider. Due to the greater demand for animals with a comfortable gait for traveling long distances and at low speeds, marching saddle mules have been selected by crossing of marching donkeys of the Pêga and Brasileira breeds with horses, preferably those of the Mangalarga Marchador and Campolina breeds [19]. Although the AA genotype, when compared to the CA or CC genotype, provides greater support for ipsilateral limb coordination, along with a negative effect on synchronized lateral diagonal movement [3], which would be of benefit to the picada gait type, in the present study we did not observe the presence of mutant homozygous AA mules or donkeys. In addition, the CA genotype was observed at a low prevalence in the donkeys (3%, 7/203) evaluated in the present study, especially when compared to the frequency of this genotype in mules (53%, 84/159). Overall, the frequencies of allele A in the groups of mules and donkeys, regardless of gait, were 0.264 ± 0.025 and 0.017 ± 0.006, respectively. Several studies have also linked the presence of the AA genotype with gait in horses [2,3,4,6,7]. However, similar to the present study, when the presence of *DMRT3*_chr23:g.22999655C>A was evaluated in a group of donkeys in India [22], only animals homozygous for the reference allele C were found. Although the number of donkeys was small, these authors suggested that this SNP has been equally distributed among the breeds of donkeys in India.

In contrast to what was observed in the present study, where the CC and CA genotypes were similarly distributed both in the mules with the batida gait (*p* = 0.484) and in the mule with the picada gait (*p* = 0.749), Manso-Filho et al. [17] found only the CC genotype in Mangalarga Marchador horses that presented a batida gait, while in animals with a picada gait, the A allele was predominant, both in heterozygosity and in homozygosity. Other authors [17,18] also observed the predominance of the C and A alleles in the Mangalarga Marchador that presented batida and picada gaits, respectively. Although more mules were genotyped in the present study (159) than were horses for *DMRT3*_chr23:g.22999655C>A by Manso-Filho et al. [17] (n = 105) or Patterson et al. [16] (n = 81), it is difficult to rule out this variant being related to gait in mules. Although the predominance of the CC genotype was observed both in the donkeys with the batida gait (*p* < 0.001) and in the donkeys with the picada gait (*p* < 0.001) gaits, the CA genotype was more frequently observed (*p* < 0.001) in donkeys with the picada gait (6/7, 86%) than in those with the batida gait (1/7, 14%). Therefore, even considering the exception of the absence of AA donkeys and the low number of CA donkeys, the greater proportion of this genotype in the donkeys with the picada gait is in agreement with other studies in Mangalarga Marchador horses [16,17,18]. Such associations for the DMRT3 SNP were verified in the Hokkaido Native Horses, a Japanese native breed, where all horses exhibiting the pace gait possessed the AA genotype for *DMRT3* whereas among horses exhibiting the trot gait, 14% also had the AA genotype for *DMRT3* SNP, indicating the potential involvement of another factor(s) along with DMRT3 in determining gait [25]. Therefore, future investigations involving donkeys and mules in Brazil may focus on exploring additional contributing factors.

## 5. Conclusions

The fact that the three *DMRT3*_chr23:g.22999655C>A genotypes have already been described in Mangalarga Marchador and Campolina horses [16,17,18], the main horse breeds involved in the commercial production of mules in Brazil, in combination with the higher percentage of heterozygous mules and the low frequency of the mutated allele in the donkeys evaluated in this study, allows us to speculate that the mutated allele observed in heterozygosity in the mules in this study was inherited, above all, from the mare used in the cross with the donkeys. The influence of the maternal factor, specifically the dam’s gait, on gait determination was assessed in a previous study aimed at investigating other potential determinants of gait in Hokkaido Native Horses. Those authors suggested that the dam’s gait does not significantly affect whether progeny with the AA genotype for *DMRT3* will exhibit a preference for pacing or trotting [25]. Contrary to our expectations, the type of gait, picada or batida, was not influenced by the *DMRT3* SNP in the mules or donkeys evaluated. In Mangalarga Marchador horses, a study has shown that unlike what happens in the picada gait (lateralized), which is influenced by the A allele, the batida gait (diagonalized) may be controlled by another group of genes [15]. Therefore, future studies can investigate genes that might be associated with gait type in mules and donkeys, such as genes that are associated with differences in metabolic changes in horses with a batida or picada gait after gait exercise [26].

## Figures and Tables

**Table 1:** PCR primers for the amplification of the *DMRT3* gene exons, 5′ and 3′ intron–exon junctions, and 5′ and 3′ UTRs in donkeys (*Equus asinus*), and for the detection of EquCab3.0 *DMRT3*_chr23:g.22999655C>A variant (OP068195.1 c.902 C>A).

Primers	Sequences	Product (bp ¹)	Annealing Temperature
JP_*DMRT3*_1	AAAGACGGGTGCCGCAT GTAGCGCTTGTGACCCTTGA	315	64 °C
JP_*DMRT3*_2	GCTTCAAGGACTGCACCT TTCTGCCCGGAAAGAACC	479	62 °C
JP_*DMRT3*_3	CTGGCTCAAGGGTCACAAG AGGCCAACTTCCGAAACC	457	62 °C
JP_*DMRT3*_4	CATTTGCCAGTGACATAGTTTGG CAAGCTGAAGGGCAGAGAAA	454	59 °C
JP_*DMRT3*_5	CAGAGACCTTCAGCGACAAA GTCATCCTCGGTGTAAAGAGAC	866	62 °C
JP_*DMRT3*_6	GCAGACTCTAGTAACGTTGTCC CCCAGCTTTCCCAAGACTATT	666	62 °C
JP_*DMRT3*_DdeI	ACAGAGACCTTCAGCGACAA GGGTTGGGGACAACGTTACT	560	60 °C

^1^ bp, base pairs.

**Table 2:** Distribution of genotypes (CC, CA, and AA) of the EquCab3.0 *DMRT3*_chr23:g.22999655C>A variant according to the phenotype (batida or picada gait) found in 159 mules.

	Gait		
Genotypes	Batida	Picada	Total
CC	46 (46%)	29 (48%)	75 (47%)
CA	53 (54%)	31 (52%)	84 (53%)
AA	ND ^1^	ND ^1^	
Total	99	60	159

^1^ ND, not detected.

**Table 3:** Distribution of genotypes (CC, CA, and AA) of EquCab3.0 *DMRT3*_chr23:g.22999655C>A variant according to the phenotype (batida or picada gait) found in 203 donkeys.

	Gait		
Genotypes	Batida	Picada	Total
CC	100 (99%)	96 (94%)	196 (97%)
CA	1 (1%)	6 (6%)	7 (3%)
AA	ND ^1^	ND ^1^	
Total	101	102	203

^1^ ND, not detected.

## Data Availability

The datasets used or analyzed during the current study are available from the corresponding author upon reasonable request.
